# Tools, Technologies and Frameworks for Digital Twins in the Oil and Gas Industry: An In-Depth Analysis

**DOI:** 10.3390/s24196457

**Published:** 2024-10-06

**Authors:** Edwin Benito Mitacc Meza, Dalton Garcia Borges de Souza, Alessandro Copetti, Ana Paula Barbosa Sobral, Guido Vaz Silva, Iara Tammela, Rodolfo Cardoso

**Affiliations:** Institute of Science and Technology, Fluminense Federal University, Rio das Ostras 28895-532, Brazil; emitacc@id.uff.br (E.B.M.M.); alessandro_copetti@id.uff.br (A.C.); ana_sobral@id.uff.br (A.P.B.S.); guido_vaz@id.uff.br (G.V.S.); iaratammela@id.uff.br (I.T.); rodolfo_cardoso@id.uff.br (R.C.)

**Keywords:** digital twin, oil and gas, systematic literature review, decision support systems

## Abstract

The digital twin (DT), which involves creating a virtual replica of a physical asset or system, has emerged as a transformative set of tools across various industries. In the oil and gas (O&G) industry, the development of DTs represents a significant evolution in how companies manage complex operations, enhance safety, and optimize decision-making processes. Despite these significant advancements, the underlying tools, technologies, and frameworks for developing DTs in O&G applications remain non-standardized and unfamiliar to many O&G practitioners, highlighting the need for a systematic literature review (SLR) on the topic. Thus, this paper offers an SLR of the existing literature on DT development for O&G from 2018 onwards, utilizing Scopus and Web of Science Core Collection. We provide a comprehensive overview of this field, demonstrate how it is evolving, and highlight standard practices and research opportunities in the area. We perform broad classifications of the 98 studies, categorizing the DTs by their development methodologies, implementation objectives, data acquisition, asset digital development, data integration and preprocessing, data analysis and modeling, evaluation and validation, and deployment tools. We also include a bibliometric analysis of the selected papers, highlighting trends and key contributors. Given the increasing number of new DT developments in O&G and the many new technologies available, we hope to provide guidance on the topic and promote knowledge production and growth concerning the development of DTs for O&G.

## 1. Introduction

The oil and gas (O&G) industry has played a central role in the global economy for over a century. Its complex exploration, production, and distribution network is vital for global energy supply. While the industry is constantly evolving with technologies and creative solutions to address the growing energy needs, it currently grapples with significant obstacles in its pursuit of operational excellence and enhanced productivity [1,2,3]. These challenges emphasize the necessity for significant innovations, disruptions to traditional methods, strategic restructuring, and a more sustainable, efficient approach.

In this scenario, the digital twin (DT) concept emerges as a tool that effectively meets the intricate demands of the O&G industry. Among the definitions presented in the literature [4,5,6,7,8], one definition from the American Institute of Aeronautics and Astronautics (AIAA) in [9] shines through: “*A set of virtual information constructs that mimics the structure, context, and behavior of an individual/unique physical asset, or a group of physical assets, is dynamically updated with data from its physical twin throughout its life cycle and informs decisions that realize value*”. This explanation underscores how the DT differs from an entity by integrating data gathering and application to support decisions that enhance value across the system life cycle. This strategy seamlessly fits with the needs of the O&G industry, where assets and complex operations are crucial, and data-informed decisions can significantly boost efficiency, safety, and sustainability [5,7].

Digital twins have gained significant traction in the O&G industry due to their ability to provide real-time diagnostics and predictive maintenance, e.g., [10], improving operational safety and efficiency. A prominent application is in the optimization of packer setting operations, where DTs can identify potential failures before they occur, integrating comprehensive models with real-time surface data to dynamically monitor and adjust completion operations [11]. This approach reduces time and operational risks while enhancing decision making by comparing real and expected data under quantitative criteria. DTs heavily rely on accurate, real-time data acquisition through advanced sensors, which are crucial for maintaining dynamic updates of virtual models. Studies show that integrating DTs with sensor data enhances monitoring and predictive capabilities by improving data fidelity and providing an anticipatory view of operational conditions, which are essential for the continuous assessment of sufficient conditions and other parameters in complex environments [12].

According to recent studies, the O&G industry faces challenges in adopting DT technology. As noted in [13], one of the obstacles is the need to approach the DT journey strategically and thoughtfully rather than a haphazard one to influence the technology’s effectiveness and outcomes significantly. In addition, real-time monitoring requirements introduce another layer of complexity, necessitating processing power, fast data transmission speed, and reliable systems [14]. Considering that the industry’s operations are intricately linked with macroeconomic and environmental factors, it is crucial to balance the strategic planning and innovative application of technology to harness digital twins’ benefits fully. This alignment is essential for achieving excellence and sustainability goals within the industry [15].

The use of digital twins (DTs) presents a variety of opportunities, such as improving efficiency, knowledge sharing, safety, and information integration across the organization [1]. Combining edge computing infrastructure and cybersecurity measures offers an advantage by providing defenses against advanced attacks on IT (Information Technology) systems [16]. Real-Time Safety Solutions (RTSS), as emphasized by [17], revolutionize personnel monitoring and safety through the use of intelligence and automation in high-risk operations. Moreover, fostering a shift toward transformation within companies is essential, and it requires effective leadership and a willingness to adopt new technologies [15].

In particular, the DTs have vast potential within the O&G industry, where real-time data analytics and virtual reality integration can transform training and operational strategies [14]. Therefore, companies do not simply update their technology when they adopt digital twins. Instead, they are strategic assets that can shape the future of operations and establish new standards of operational excellence in the industry.

To grasp the potential of DT technologies in the O&G industry, researchers must explore this cutting-edge advancement in depth. Review studies like those mentioned in [16,18,19,20] have established the groundwork by showcasing the applications and advantages of DTs in enhancing operational efficiency and decision-making processes.

While our objectives have some similarities to the in-depth review by [18], which discusses research trends, opportunities, and challenges in digital twins for the O&G industry, our main focus is on the technical aspects inherent in the development life cycle of a digital twin within the O&G domain. Our findings and interpretations are also distinct and complementary to other studies, such as that by [19], which explores the role of digital twin modeling in real-time production and its integration within hydrocarbon enterprises. Similarly, the research by [20] provides perspectives on the transformative role of artificial intelligence in drilling and completion. Finally, the contribution from [16] elaborates on the synergy between digital twins and cloud-edge computing paradigms in the O&G industry, outlining the challenges and advantages of these integrative solutions. The framework proposed by [21] focuses on virtual representations in the energy sector, while our work also consolidates the study with a framework that considers the challenges of the O&G industry, such as real-time monitoring and optimization in drilling, offering a complementary yet distinct approach.

Nevertheless, existing studies have predominantly focused on the implications and technological aspects of DTs. There remains a need to explore the specific methodologies employed in the development of DTs and to conduct a comprehensive analysis of their evolutionary stages. This significant gap in the current literature, particularly in the context of the O&G industry, underscores the necessity for a systematic investigation that addresses the unique challenges and requirements involved in the development and life cycle of DTs from their initial creation to their ongoing application within this sector.

The main purpose of this paper is to explore the methods and techniques employed in developing digital twins in the O&G industry through a systematic literature review (SLR). It covers the phases, tools, and technologies involved in their development from inception to application and continuous upkeep in real-world scenarios. The objective is to enhance the existing knowledge pool by offering perspectives that can help researchers and practitioners enhance the use of twins, promoting innovation and productivity within the O&G industry.

The article’s structure is as follows: Section 2 presents the methodological approach used in this study, including data collection and analysis techniques. Section 3 offers a quantitative analysis of the selected literature, highlighting key trends and contributors. Section 4 explores the various aspects of digital twin technology in the oil and gas industry using the SLR. Section 5 introduces a conceptual framework for its development. Finally, Section 6 provides the conclusions.

## 2. Methodological Approach

This paper describes a systematic literature review following the guidelines recommended by [22] for conducting a thorough SLR. In addition to this approach, we have utilized the PRISMA framework [23], which includes a 27-item checklist to ensure transparency and consistency in SLRs. We have also employed Parsifal, a dedicated SLR support platform, to help establish review protocols and facilitate the efficient search, selection, and synthesis of relevant papers. Furthermore, we have incorporated insights from methodologies presented by [24,25], paying particular attention to their pre-search stages.

Formulating our research questions (RQs) was crucial for guiding our SLR. These RQs were developed prior to the comprehensive search and were informed by an initial exploration of the existing literature, covering key topics related to DTs and the O&G industry [16,18,19,20,26,27,28]. This preliminary investigation allowed us to identify key themes, gaps in the literature, and relevant keywords, ensuring a comprehensive and focused review. The primary research questions addressed in this study are outlined below:

RQ1: What are the key trends and patterns in developing and applying DTs within the O&G industry, as shown by publication profiles, authorship patterns, and the geographic distribution of research contributions?
RQ2: What are the current methodologies used in developing DTs within the O&G industry, and how do they contribute to constructing and conceptualizing these digital counterparts?
RQ3: What specific objectives drive the implementation of DTs in the O&G industry, and how do these objectives shape the design and application of DTs?
RQ4: How are data acquisition processes, particularly through sensor technologies, designed and implemented within the O&G industry to support the development and operationalization of DTs?
RQ5: What tools and technologies are utilized in the digital development of assets for DTs, and how do these resources facilitate the creation of accurate and dynamic virtual replicas of physical assets?
RQ6: What are the predominant data analysis and modeling approaches used in the context of DTs, and how do they enhance the ability of these systems to predict, optimize, and analyze data for improved decision making?
RQ7: Through what metrics and methodologies are DTs evaluated and validated within the O&G industry to ensure their effectiveness and representational accuracy?
RQ8: What are the preferred software solutions and computational platforms for deploying DTs in the O&G industry, and how do these choices reflect the industry’s specific needs and challenges?

**Figure 1:**
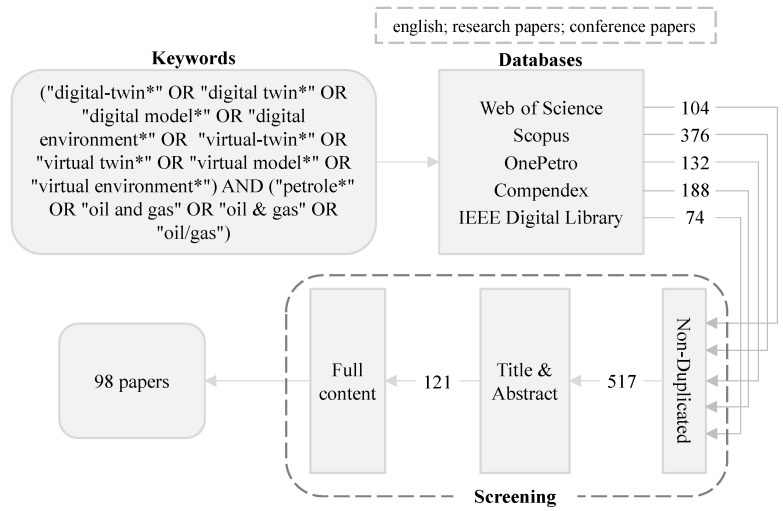
Article selection framework (Note: the asterisk represents a wildcard for zero or more characters in the search terms).

These research questions shaped our search and selection criteria, ensuring that our review was comprehensive and focused on relevant studies. From the initial exploration of existing literature, we gathered lists of essential keywords, relevant research databases, and commonly studied topics within the DTs and O&G. A visual representation of the identified research databases and keywords can be seen in Figure 1. These articles were chosen based on their titles, abstracts, and keywords.

The research involved using five well-known databases (see Figure 1), which were selected for their coverage of multidisciplinary, engineering, and domain-specific research in DTs and O&G. Scopus, for example, is a comprehensive multidisciplinary database containing around 15,000 peer-reviewed journals from over 4000 publishers. Its broad scope ensures access to studies from various scientific fields, which is crucial given the multidisciplinary nature of DT research. Similarly, the Web of Science Core Collection, which encompasses approximately 10,000 peer-reviewed journals, was chosen due to its long-standing reputation as a reliable citation database across all scientific domains [29]. In addition to these multidisciplinary resources, we selected domain-specific databases used in SLRs related to O&G: Compendex, IEEE Digital Library, and OnePetro. Compendex offers extensive coverage of engineering disciplines, making it particularly valuable for studies associated with the technical aspects of DTs. The IEEE Digital Library was included because it is a crucial source of high-quality, peer-reviewed technical literature in engineering, computer science, and related fields. OnePetro, a specialized database for O&G, was critical to ensure the inclusion of highly relevant industry-specific research.

While we acknowledge the existence of additional databases, these five were selected for their balance between multidisciplinary coverage and specificity to DTs and O&G research. This selection allowed us to conduct a thorough, targeted review of high-impact, peer-reviewed articles. Our search began on 1 August 2023 and concluded on 31 December 2023. We identified 1300 articles from 2018 (the year the term “digital twin” first emerged in O&G literature [18]) through to 2023. From this pool, we prioritized non-duplicated, English-language articles from peer-reviewed journals and conferences. Our assessment involved a three-stage screening process. Initially, we evaluated titles, discarding those that did not align with our study’s focus. Subsequently, articles were further filtered based on their abstracts and full content, leading to the selection of 98 articles. The screening process was comprehensive, encompassing evaluations at multiple stages as detailed in detailed in Table 1. It is important to note that each step was carried out with awareness of the criteria set for the succeeding stages. Also, since the data collection from the papers happened parallel with the paper acquisition and reading, not all papers were included in all analyses we performed.

This methodological approach ensures a rigorous and comprehensive review of the literature on DTs in the O&G industry, providing valuable insights into current trends, methodologies, and technological advancements. Based on this foundation, we will address the main research questions in the following sections, thoroughly examining the role of DTs in the O&G industry.

## 3. Quantitative Analysis

In this section, we will conduct a bibliometric analysis to explore the publications on DT in the O&G industry, explicitly addressing Research Question 1 (RQ1). Our analysis aims to address several related research questions, including the geographical distribution of research efforts, author affiliations, and the corporate entities most frequently associated with DT studies. We will quantitatively assess the articles to create a publication profile highlighting the leading countries in DT research, the proportion of articles authored by corporate versus academic entities, and the stages of DT development discussed in the literature.

**Figure 2:**
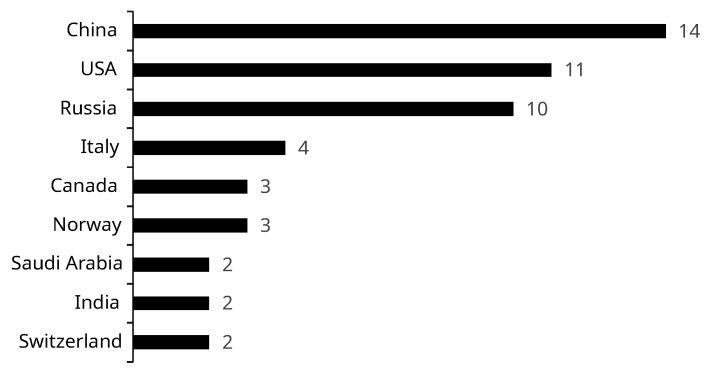
Countries with the most publications.

A bibliometric analysis quantifies the articles under examination and presents the publication profile in the respective field. China, the USA, and Russia emerge as the leading countries of the first authors, with 14, 11, and 10 articles, respectively. However, approximately 34.44% of the first authors did not have their nationality identified in the article. Figure 2 displays all the countries with at least two articles each.

**Figure 3:**
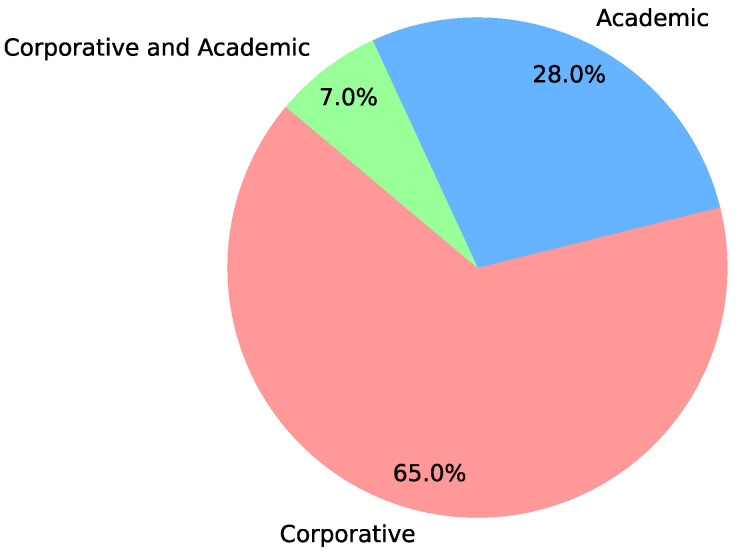
Profile of authorship in publications.

Regarding the authors, 65% of the articles are authored solely by corporate entities, while 28% originate only from academia. Approximately 7% of the articles are the result of collaborations between universities and industry. These statistics underscore the emphasis on the practical application of digital twins and reveal that the main sources of scientific articles in this systematic literature review are conferences and seminars. This distribution also explains why there is uncertainty and lack of consensus on digital twins, as their practical application and implementation have been more prominent than theoretical developments. The authorship profile of the articles is depicted in Figure 3.

**Figure 4:**
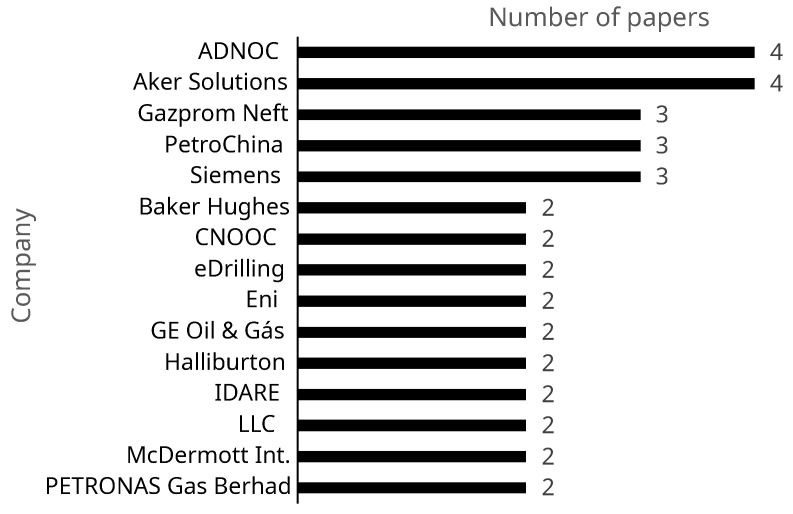
Companies with the most appearances in articles.

The emphasis on practical application is also reflected in the large number of companies being the subjects of the authors’ studies. About 72% of the articles specify which companies are the focus of their study. The primary companies highlighted in this dataset are the Abu Dhabi National Oil Company (ADNOC), Aker Solutions, Gazprom Neft, PetroChina Company, Siemens, Baker Hughes, CNOOC, eDrilling, Eni, GE Oil & Gas, Halliburton, IDARE, LLC, McDermott International, and PETRONAS Gas Berhad. Figure 4 illustrates the main companies and the number of articles dedicated to each.

**Figure 5:**
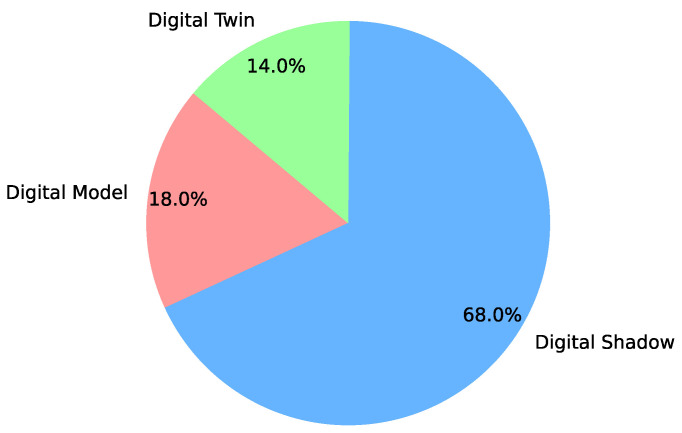
Different stages of digital twin development.

The concept of digital twins, as presented in various articles, has several interesting aspects worth noting. Many authors, including [30,31,32], follow the evolutionary progression of a digital twin, which they define as a model capable of real-time data integration and influencing the operation of the physical asset through a bidirectional flow of information. The preceding stages of a digital twin include the digital shadow and digital model. The digital model represents the initial stage, where there is no bidirectional flow or real-time data integration, comprising only batched data integrated into a geometric model. The digital shadow serves as an intermediate level, featuring real-time data integration but lacking bidirectional information flows. This analysis does not delve into the examination or proposition of similar stages. Nevertheless, considering these definitions, it was observed that 68% of the articles focus on digital shadow implementations, 18% on digital models, and only 14% on actual digital twins (refer to Figure 5), highlighting the absence of consensus on the characteristics of a digital twin.

**Figure 6:**
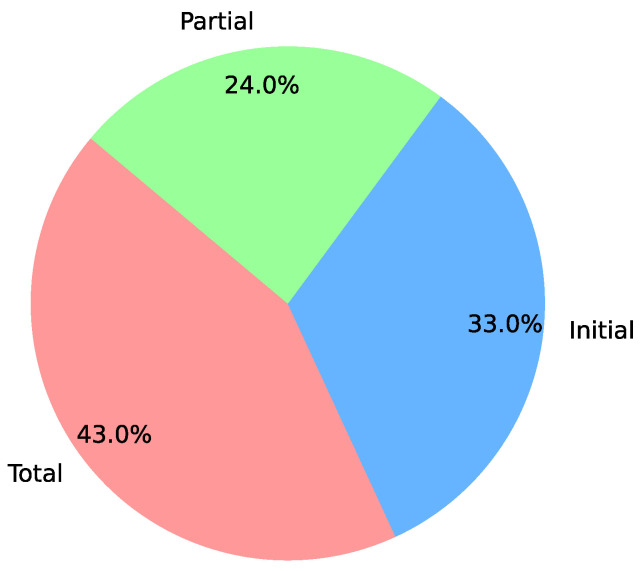
Stages of implementation in publications.

Regarding the implementation stage of the digital twins described in the works, full implementation was noted in only 43% of the works with approximately 57% at initial or partial implementation stages (see Figure 6). This observation reveals that the literature not only comprises successful implementations but also describes ongoing implementation processes.

**Figure 7:**
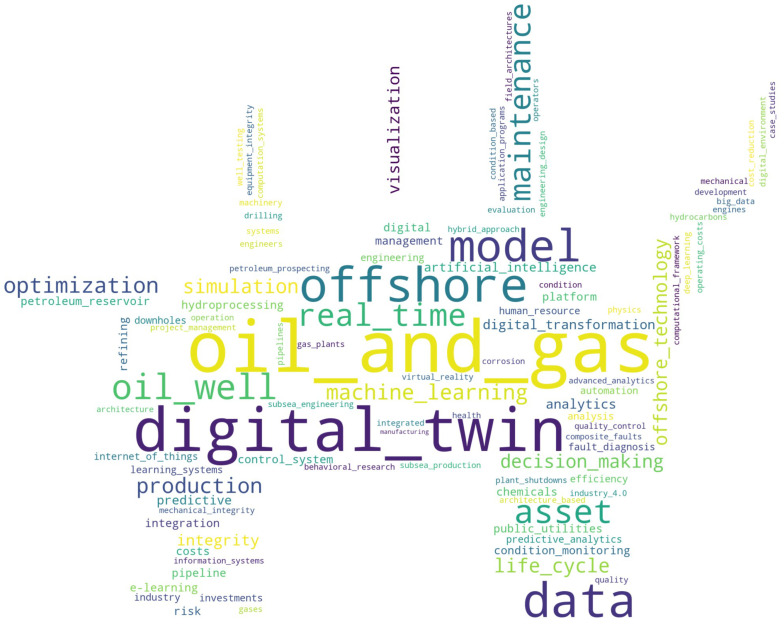
Word cloud of primary keywords from the selected articles.

Finally, Figure 7 presents a word cloud illustrating the primary keywords from a systematic literature review on digital twins in the oil and gas industry. This visualization highlights key research trends and technologies by depicting the frequency of terms across the reviewed articles. Prominent terms such as “machine learning”, “real time”, “optimization”, and “simulation” underscore the focus on advanced technologies enhancing operational efficiency and decision making. Additionally, words like “offshore”, “asset”, and “maintenance” point to specific applications of digital twins, reflecting their critical role in the predictive maintenance and asset management in offshore operations.

## 4. Qualitative Analysis

This section explores the development of DTs in the O&G industry, addressing various research questions (RQs). It is divided into seven areas: frameworks for DT development (RQ2), implementation objectives (RQ3), data acquisition (RQ4), asset digital development (RQ5), data analysis and modeling (RQ6), DT evaluation and validation (RQ7), and DT deployment (RQ8). It begins by identifying methodologies for DT development, emphasizing the early stage of this technology within the industry. The discussion extends to implementation objectives, categorized into monitoring, control, management, optimization, and prediction, highlighting the strategic intent behind deploying DTs to enhance operational efficiencies and predictive capabilities.

The narrative further delves into data acquisition techniques, emphasizing the importance of sensor technologies in capturing critical operational data, which is essential for the accuracy and utility of DTs. Asset digital development is another key focus, exploring the use of various modeling tools that enable the creation of highly accurate and dynamic DTs, bridging the physical and digital realms.

Data analysis and modeling are highlighted as crucial for interpreting the vast amounts of data generated, using sophisticated machine learning and simulation techniques to predict and optimize performance. The section on DT evaluation and validation stresses the importance of rigorous testing methodologies to ensure the reliability and representativeness of DT models, which is a critical step for their successful application. Finally, the deployment of DTs is discussed, noting the preference for specialized software solutions tailored to the unique demands of the O&G industry.

### 4.1. Methodologies for Digital Twin Development

Out of the 98 articles reviewed, we found a variety of strategies and approaches for designing digital twins but with limited levels of detail. Some articles prioritize showcasing the final architecture diagram, structure, or model of the digital twin over explaining the steps for building it.

Only 35 articles in the sample outlined methodologies or frameworks for developing the DTs. Within this group, ten articles provided descriptions or engaged in more direct discussions about the methods used to build the specific DT solution [33,34,35,36,37,38,39,40,41,42]. Furthermore, six of these studies scrutinized the step-by-step construction of the digital twin in question, providing a comprehensive view of the main stages [34,36,37,38,41,42].

The diversity of approaches observed can be explained by certain characteristics. One factor contributing to this diversity is the emerging interest in researching and applying DTs in the O&G industry, which may result in a lack of established reference methods. Additionally, many articles identified from the O&G industry conferences focus more on presenting the final solution and its results rather than discussing the methodological steps for designing the DT solution [35,36,38,39]. The sample revealed variations in DT maturity levels and application objectives, resulting in different design methods.

To summarize, the main methodological aspects found were organized into categories, which made it clearer how digital twins are developed. This encompasses aspects ranging from system architecture and data handling to modeling, validation, and optimization. Table 2 shows the primary aspects outlined in the analyzed articles.

In the first two stages, it is essential to use modeling techniques that form the foundation of digital twin development. These techniques allow for the creation of accurate virtual representations. The first technique is physics-based modeling, which utilizes mathematical equations and principles to simulate the behavior of physical systems [34,35,37,38,39]. This method ensures high-fidelity representations but may require extensive computational resources. The second technique is data-driven modeling, which uses historical and real-time data to construct DTs [34,36,42]. Machine learning algorithms analyze data patterns to predict behavior, offering scalability and adaptability. It is essential to analyze existing sensors and their sufficiency because sensor integration is crucial in capturing real-world data and updating digital twin models in real time.

DTs should support situation awareness stages: perception, comprehension, and projection [34]. Perception involves accessing sensor and contextual data and emphasizes the importance of data synthesis for usability. Comprehension entails assisting users’ memory and creating models of situations, highlighting the role of data management in linking past experiences with current situations. Projection necessitates simulation capabilities for anticipating future system states.

DTs facilitate operational optimization in oil and gas by integrating artificial intelligence and genetic algorithm-based optimization techniques [43]. Simulation and visualization techniques enable the analysis and interpretation of digital twin data, facilitating decision making and optimization. Integration with artificial intelligence and analytics enhances the capabilities of digital twins, enabling advanced predictions and optimizations. Techniques like virtual prototyping and 3D visualization allow for testing design iterations and scenarios in a risk-free digital environment, reducing time-to-market and costs, and rendering digital twins in three-dimensional space, offering intuitive insights into complex systems and facilitating stakeholder understanding. Predictive analytics and optimization algorithms utilize historical data and machine learning algorithms to forecast future behavior and trends, aiding proactive decision making and risk mitigation, and applying optimization techniques to digital twin models, identifying optimal configurations and strategies for improved performance.

The validation of digital twin models involves testing their predictive power and accuracy using real-world data, often through statistical analysis and comparison with existing systems [43,44]. Optimization techniques, such as multiobjective optimization, are applied to refine digital twin models and improve their performance in achieving specific objectives, such as reducing downtime and increasing efficiency [43]. Training and testing are necessary to ensure the performance of models against unplanned shutdown events. They use historical data and validate them with real-time operational data as well as update models iteratively based on new data and operational insights to improve performance and accuracy.

Finally, a life cycle management method must ensure the continuous improvement and relevance of digital twins throughout their operational lifespan. This category’s strategies include integrating diverse data sources and regularly updating digital twin models to reflect real-world changes, maintaining accuracy and reliability, and establishing feedback loops between physical assets and their digital twins, enabling iterative improvements and adaptive responses to evolving conditions.

### 4.2. Implementation Objectives

The O&G industry implements DTs to meet operational and strategic demands. These advanced digital models are designed with specific goals in mind, such as process optimization, enhanced monitoring, data visualization, improved operational efficiency, anticipating failures, proactive maintenance, and evidence-based decision making. These objectives can be categorized into three main types: Monitoring, Control, and Management (featured in 9 articles), Optimization of Outcomes and Resources (featured in 12 articles), and Prediction and Detection of Abnormalities (featured in 10 articles).

#### 4.2.1. Monitoring, Control, and Management

A study by [45] noted that the management of real-time data can be improved by using 3D holographic projections, which integrate the physical environment with the digital. This suggests a focus on data processing accuracy and efficiency. Ref. [39] highlights digitalization in directional drilling planning, emphasizing the real-time management of the drilling process and the trend toward data-driven decision making. Additionally, ref. [46] adds that drilling and oil exploration management are relevant applications for DTs, indicating their role in optimizing existing processes. Ref. [47] emphasizes the ability to monitor drilling operations.

Ref. [48] discusses the need for strategies addressing the management and maintenance of equipment and infrastructure in asset management. The proposal by [49] suggests a knowledge-based DT prototype for the upstream sector, advancing the application of industry-supported IoT technologies and promoting integration between the operational scenario and DT models. Moreover, ref. [50] highlights the importance of enhancing the integrity and efficiency of CO_2_ piping systems, showing concern for sustainability and environmental impact.

Furthermore, ref. [51] emphasizes the crucial role of simulation in operational training, where analytical and numerical models are used to replicate drilling conditions, preparing teams for various contingencies. Ref. [52] addresses the improvement of the quality of operation of industrial control systems and the reduction of incidents, focusing on the safety and reliability of production systems. Finally, in a study by [53], planning and executing inspections were mentioned, and the combination of DTs with artificial vision technologies was noted to facilitate accurate inspection and maintenance.

#### 4.2.2. Optimization

In the context of optimization in the O&G industry, DTs have played a critical role. Generally, optimization is a primary focus in the development of DTs, as noted by [34,54]. These optimization efforts are applied in various areas, from pipeline design to energy efficiency. Data quality control and real-time optimization are essential for drilling operations and are being enhanced by DTs [55].

An artificial intelligence-driven engineering calculation system is referred to as a crucial tool for optimizing pipeline design by [36], reflecting the ongoing effort to save engineering time and accommodate economic uncertainties. On the other hand, ref. [48] highlights improvements in drilling efficiency, including an increase in penetration rate and a reduction in non-productive time with the goal of optimizing well placement through planned geological targets. The reduction of well tortuosity and reduced dependence on operator experience suggest a shift toward autonomous systems. The focus of [56] is the development of a new Field Development Planning (FDP) to maximize investment return, underlining the importance of strategic optimization in long-term planning.

Ref. [57] discusses the benefits of using DTs for drilling activity efficiency, while [58] points to optimizing performance in natural gas treatment stations, focusing on maximizing hydrocarbon recovery. In terms of resource utilization, reducing energy consumption, especially in crude oil distillation units, is a critical aspect of resource optimization identified by [59]. This goal aligns with the need to reduce operational costs and improve energy efficiency. Additionally, ref. [60] mentions drilling automation technology and real-time monitoring to reduce non-productive time, highlighting the commitment to operational efficiency and tool longevity.

Finally, ref. [61] emphasizes improvements in sensor calibration and the accuracy of fluid analyses, which are technical optimizations that directly impact the accuracy of drilling and production operations.

#### 4.2.3. Prediction

In the O&G industry, the use of DTs for prediction and anomaly detection is crucial for maintaining operational integrity. According to [62], DTs are used to diagnose and resolve minor failures in underwater production control systems, which is a critical application due to the complexity and risk involved in these operations. Ref. [40] states that DTs not only identify equipment failures in real time but also provide maintenance and repair forecasts, allowing for proactive interventions to prevent failures.

As noted in [63], asset integrity is a constant concern, and continuous monitoring through DTs allows for effective management, leading to uninterrupted operations and extended equipment lifespan. Hybrid data analysis, presented in [64], is an innovative methodology that combines sensor data and simulation models to monitor equipment conditions in real time, using performance degradation metrics and anomaly detection as key indicators.

Comprehensive digitalization, as outlined in [65], forms the foundation for a risk-based inspection strategy, enabling the evaluation of anomalies in operational environments. Additionally, ref. [66] proposes a specific model for anomaly detection, which is essential for preventing operational incidents. According to [67], DTs are crucial in detecting issues such as well leaks, while [68] emphasizes their application in identifying leaks in natural gas pipelines.

Finally, according to [63], DTs are vital for diagnosing failures in submarine production control systems, ensuring the reliability of processes that would otherwise be inaccessible and prone to risks. This application showcases the ability of DTs to operate in challenging environments, providing valuable insights for maintaining safety and efficiency.

### 4.3. Data Acquisition

In the O&G industry, acquiring sensor data is essential for many operations, such as project planning, asset monitoring and maintenance, and managing offshore platforms and pipelines. This process involves using various technologies and systems to ensure the efficient collection, transmission, and analysis of data that is critical for operational decisions and strategic planning. According to [69], integrating Industrial Internet of Things (IIoT) technologies is a common theme across these applications. For example, database connectors like KEPWARE enable seamless connections with SCADA (Supervisory Control and Data Acquisition) systems, allowing real-time data flow from the field to the database systems. These connectors make it easier to collect and integrate data from different sources, including corporate and internal databases, thus supporting the extensive data needs of DTs and other analytical tools.

The industry uses advanced data acquisition systems that integrate sensor data with other operational data sources. These systems utilize protocols and technologies such as IIoT, SCADA, and cloud computing to ensure the efficient collection, transmission, and analysis of data [63]. The use of cloud-based platforms and data lakes for storing and processing these data highlights the shift toward more scalable and flexible data management solutions, enabling the aggregation of vast amounts of data from diverse sources [70].

As detailed in [36], most digital twins in the O&G industry heavily rely on real-time data acquisition. Creating these digital replicas requires a strong data infrastructure capable of handling inputs from various data sources, including sensors, to accurately reflect the current state of physical assets and predict future performance. Sensors capture critical data points that feed into systems designed to enhance operational efficiency, safety, and productivity [34].

In pipeline design and integrity engineering, data ingestion systems use information from inline inspections and other sensor-based measurements to predict operational integrity. The sophisticated architecture supporting these systems includes connections through various protocols such as MQTT (Message Queuing Telemetry Transport), OPC UA (Open Platform Communications Unified Architecture), and REST API (Representational State Transfer Application Programming Interface), among others. These connections illustrate the complex data ecosystem required to support O&G operations [36]. Additionally, the use of LiDAR (Light Detection and Ranging) sensors for mapping and monitoring, like assessing pipeline integrity or construction quality, highlights the technological advancements being adopted in the field. These sensors provide high-resolution data critical for maintaining the long-term integrity and safety of O&G infrastructure [71].

As mentioned by [37], sensors are not only used for external monitoring but also for wellbore environments. In these environments, temperature, pressure, and flow sensors are deployed to detect leaks and provide a detailed understanding of well conditions. This understanding is crucial for effective well management and incident prevention in the O&G industry, which operates in a complex and dynamic environment. Monitoring various parameters and variables through sensors is critical for ensuring operational efficiency, safety, and informed decision making across multiple sectors within the industry, including exploration, production, drilling, and asset maintenance. This involves a wide array of sensors collecting data on different variables.

In exploration and drilling, sensors such as those for Logging While Drilling (LWD) provide real-time data on the geological properties encountered during drilling operations. These data are crucial for adjusting drilling strategies and minimizing risks associated with drilling in complex formations [72]. Additionally, accelerometers and gyroscopes are used on drilling risers to monitor vibrations, helping prevent structural failures that could lead to operational downtime or environmental disasters [73].

Sensors play a vital role in implementing predictive maintenance strategies for asset performance and maintenance. They monitor equipment conditions, detect early signs of failure, and support decision-making processes that significantly reduce operational costs and enhance safety [40]. Integrating sensor data with DTs allows for the simulation and analysis of asset performance under various conditions, facilitating the proactive maintenance and optimization of operations [69].

Table 3 shows a detailed overview of sensor applications in various operational areas of the O&G industry. Most research focuses on monitoring and maintaining assets, such as [71,74]. The frequent measurement of parameters like pressure, temperature, and flow rates in multiple studies indicates their crucial role in ensuring asset integrity and operational safety. In drilling operations, sensor technologies aim to improve precision and reduce risks, as shown in studies like [72], which demonstrate the use of sensors for real-time geological insights. Exploration and reservoir management research, such as [49], highlights the adoption of IoT devices and LWD sensors for advanced data analysis. Efforts to optimize production, exemplified by entries like [75], demonstrate the industry’s increasing use of sophisticated data integration tools to enhance efficiency.

### 4.4. Asset Digital Development

Digitalization, enabled by technologies and tools, serves as a strong foundation for creating virtual environments that accurately replicate entities, behaviors, and interactions in the physical world [86]. Digital models, as indicated by [19], comprise geometric, physical, behavioral representations, and rule models. Geometric modeling tools define the shape, dimension, position, and interconnections among elements, enabling structural analyses and production process planning [86]. This is exemplified in [87], where 3D Computer-Aided Design (CAD) is used to visualize the appearance of underwater equipment, as well as production and health states to assess the system’s condition, calculate the remaining lifespan, and provide an appropriate maintenance plan. In [33], CAD tools, fueled by sensor data, are employed to depict the structure and function of an element, supporting system knowledge extraction.

Physical modeling tools construct virtual models with precise physical characteristics, facilitating the analysis of real entities’ physical states. By creating geometric models that represent objects and systems’ physical properties, these tools enable the simulation and analysis of scenarios such as structural behavior, fluid flow, and asset performance [86]. In this context, ref. [19] introduces the ANSYS FEA finite element analysis software to generate real-time states for digital models and incorporate performance degradation factors. Simulink is also mentioned for creating models based on the physical world with domain-specific modeling tools. As highlighted by [86], Simulink’s modeling approach focuses on various components, including mechanical, hydraulic, and electrical systems.

Behavioral modeling tools enable the creation of models responsive to external factors and disturbances, enhancing the performance of DT’s simulation service. By integrating dynamic system behavior into virtual models, these tools enable an analysis of how different scenarios and events may impact system performance [86]. In [34], AMESim is used to model the dynamic and transient behavior of a double-acting oil well pump through numerical simulation based on computational fluid dynamics (CFD). Table 4, developed by [86], summarizes the leading technologies for asset digitalization.

### 4.5. Data Integration and Preprocessing

Data integration and preprocessing are critical for the development and operation of DTs in the O&G industry. These processes guarantee the accuracy, relevance, and utility of DTs, ensuring they accurately reflect the actual operational conditions of physical assets. For example, during deep-sea oil exploration, equipment often generates tens of sensor data points per second, highlighting the need for technologies that can manage large data volumes for real-time analysis and the effective application of machine learning models.

Data integration in DTs for the O&G industry can utilize the Wellsite Information Transfer Standard Markup Language (WITSML) to facilitate communication between different software applications, as shown in works [47,88]. This standard is interesting for the functioning of operation centers, easing real-time data capture for the DT and providing a standardized and accessible database for stakeholders in the O&G industry.

Additionally, cloud technology and REST APIs (Representational State Transfer Application Programming Interfaces) play a significant role in data integration, as demonstrated in work [35]. These technologies enhance communication and interoperability between various systems and platforms. REST APIs are particularly important for data integration because of their flexibility, simplicity, and capability to support web services, allowing DTs to efficiently access and integrate data from diverse sources in a standardized way.

The adoption of cloud technology is a common practice in many initiatives [35,36,63,69,84,89], offering a unified platform that simplifies scalability, flexibility, and cost-effectiveness in data integration processes. This helps organizations to bypass traditional hardware and infrastructural challenges, fostering more agile and adaptable data integration strategies.

Regarding data preprocessing, technologies such as Apache Spark [90,91], Apache Flink [83], and Apache Kafka [91] are highlighted. Spark and Flink offer robust batch and real-time data processing capabilities, facilitating scalable algorithm execution. Kafka is essential for real-time data capture and transmission, employing a publish/subscribe model to enable the efficient and decoupled communication among system components. The use of these technologies in a distributed architecture is exemplified in work [83], allowing for the dynamic instantiation of processes and the adjustment of processing capacity as needed.

### 4.6. Data Analysis and Modeling

The literature discusses various tools and approaches used for data analysis and modeling in the development of digital twins. However, there is no comprehensive specification of all the tools used, as different case studies may adopt different sets of tools based on specific needs and objectives. The tools used to process and analyze data in the context of oil and gas enable the generation of insights, data analysis, anomaly detection, forecasts, and optimization. These include machine learning, numerical simulation, neural networks, and genetic algorithms.

In terms of providing insights, refs. [48,70,92] highlight the use of data visualization tools for interpreting and analyzing data collected by the DT, with “Intelie LIVE” noted in [83] as a principal platform for real-time, large-volume data visualization and monitoring.

For data analysis, the study in [49] applies correlation for pattern analysis, whereas [93] uses Partial Least Squares Regression (PLS) for assessing operational risk factors in bitumen extraction processes.

Anomaly detection methods vary, with [38] employing machine learning (model unspecified), and [87] using a Bayesian neural network. Principal Component Analysis (PCA) for dimensionality reduction alongside a deep neural network Autoencoder is utilized in [44,77] for anomaly detection. The unsupervised approach MTAD-GAN (Multivariate Time-series Anomaly Detection with Generative Adversarial Networks) is introduced in [94].

Forecasting methodologies are diverse; ref. [36] references models such as Boosting, ANN (Artificial Neural Networks), LR (Logistic Regression), RF (Random Forest), SVR (Support Vector Regression), and SVR-RBF for failure prediction. Machine learning techniques like LSTM and reinforcement learning for real-time anomaly prediction are mentioned in [63], while [37] refers to machine learning for historical matching and forecasting without specifying the models. Other forecasting approaches include machine learning for the predictive analysis of asset integrity in [69], for predicting unplanned equipment shutdowns in [63], a multivariate Convolutional Neural Network (CNN) for prescriptive maintenance in [41], and numerical simulation via the Finite Volume Method for reservoir simulations in [84]. Direct integration with the database and the “history matching” technique enable real-time updates of data and models, which are crucial for the development of forecasting plans.

In optimization, ref. [60] notes the use of machine learning for drilling data modeling to improve processes and efficiency. A combination of a neural network and genetic algorithm for asset process optimization is discussed in [43] with neural networks mentioned in [34,72,95] for similar purposes. Ref. [96] highlights Kalman filters and neural networks for problem detection and process optimization, whereas [75] introduces a Real-Time Optimization System (ROS) for diagnostics and decision making. A linear programming model for reducing energy consumption in a crude distillation unit is presented in [59].

### 4.7. Digital Twin Evaluation and Validation

The effectiveness and representational power of DTs are essential for their application in various fields. Accurately and reliably assessing DT performance is crucial to ensure their usefulness and reliability. Table 5 provides metrics and approaches used to evaluate the effectiveness and representational power of DTs.

It is important to note that the choice of the most appropriate metric or evaluation approach depends on the specific goal of the DT and the application in question. It is crucial to consider factors such as the nature of the physical asset, the granularity of the model, and the available resources. Combining different metrics and approaches can offer a more comprehensive assessment of the effectiveness and representational power of the DT.

### 4.8. Digital Twin Deployment

This section analyzes the main tools used for deploying DTs based on SLR. Table 6 details the DT solutions utilized in these studies. These tools are commercial, and one column in the table specifies the application area. The first six solutions are more general and are primarily intended for industrial use across various applications. In contrast, the last four solutions are specifically oriented toward the O&G industry, indicating a specialization in this industry.

Despite the availability of better-known and more general DT solutions from companies such as Microsoft, Amazon, Oracle, and NVIDIA, there is a preference among the few works employing digital twin software for tools specific to the O&G industry. This trend suggests a significant demand for specialized solutions capable of meeting the specific requirements of this industry even in the presence of more comprehensive platforms.

According to [19], there are limited DT applications in the O&G industry. An example of these solutions is FieldTwin (https://www.futureon.com/fieldtwin, accessed on 20 January 2024), which focuses on subsea applications. These specialized solutions are optimized for the subsea environment and/or oil production, providing functionalities, sector-specific data integration, and visualizations tailored to these complex scenarios.

It is also important to note that in addition to DT solutions, software like WellFlo (from Weatherford) enables simulations of oil fluid flows, as demonstrated in [75]. These tools contribute to the development and utilization of DTs. Moreover, software developed by oil service companies, such as Schlumberger, complements these solutions by providing advanced modeling and simulation capabilities, as indicated in [35,75].

## 5. Discussion of Systematic Review Findings

**Figure 8:**
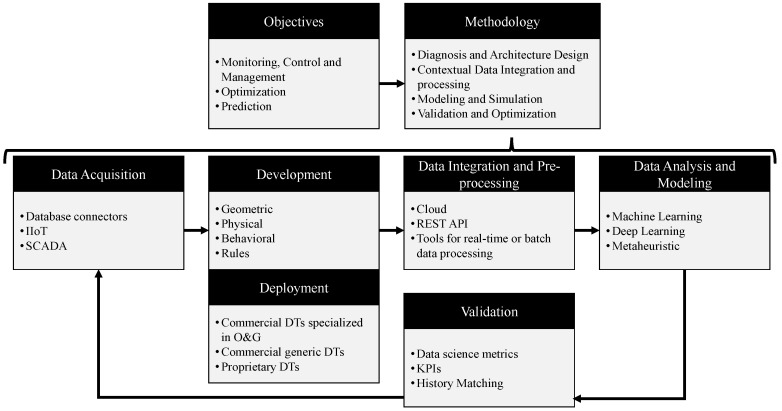
Conceptual framework for digital twin development in O&G industry.

This section summarizes the findings from the qualitative literature analysis on DT development within the oil & gas industry. Our framework, illustrated in Figure 8, integrates DT concepts and components identified in various studies, creating a comprehensive methodology tailored for the O&G industry. This approach emphasizes the industry’s need to process large volumes of data in real time. It underscores the importance of specialized tools for data manipulation in the stages of data acquisition, integration and preprocessing, and data analysis. Furthermore, the emphasis on development and deployment stages highlights the importance of flexible digital models, indicating a substantial investment in building industry-specific models. The validation stage will be integrated into the entire process to ensure that the digital twin accurately represents the physical model as it evolves.

The reviewed articles mainly focus on practical digital twin implementations rather than theoretical discussions. They present actively used tools and methodologies with a specific focus on the O&G industry. The framework emphasizes important concepts for designing and deploying models involving complex equipment that operates continuously, is prone to failures, and is exposed to extreme conditions. This approach aligns with the study’s objective outlined in the Introduction: to explore the methodologies for creating digital twins and examine the stages, tools, and technologies involved from their inception to ongoing maintenance. This approach aims to provide a comprehensive understanding and improve the practical application of digital twins within the O&G sector, enhancing operational efficiency and sustainability.

This framework significantly contributes to the systematic literature review by addressing the identified gap in the literature concerning the specific methodologies and developmental phases of DTs. Many existing studies primarily explore the benefits and applications of digital twins, often overlooking the detailed methodologies and structured processes involved in their development. This oversight creates a gap in understanding how DTs are systematically designed, implemented, and maintained. Our framework explicitly targets this gap by providing a comprehensive focus on the methodological frameworks and the DT developmental life cycle, thus filling a crucial void. It offers a structured roadmap that guides the implementation and maintenance of DT projects, ensuring a thorough understanding of each phase from inception to continuous upkeep.

This framework has theoretical and practical implications that deserve attention, since it guides designing and developing DT solutions in the O&G field. It acts as a high-level checklist among the numerous simulation and data analysis solutions developed to date. The industry has various approaches without a clear consensus, so our framework encapsulates the essential components and stages. It serves as a reference point for researchers and companies aiming to optimize DT applications, drive innovation, and improve efficiency within the O&G industry.

## 6. Conclusions

In this study, we conducted a thorough review of the existing literature to investigate the tools, technologies, and frameworks employed in developing and implementing DTs in the O&G sector. Our analysis showed significant trends and patterns, indicating a growing interest and investment in this area. The research contributions and publication profiles revealed a global effort with significant input from both academic and industrial sectors. This underscores the cooperative approach to utilizing DTs to improve operational efficiency, safety protocols, and decision-making procedures within the O&G industry.

The qualitative analysis focused on the methodologies and objectives of DT implementation. This revealed a wide range of development frameworks, emphasizing the industry’s shift toward a more data-driven approach by using IoT, machine learning, and cloud computing to create real-time models of physical assets. The importance of data acquisition through sensor technologies for ensuring the accuracy and reliability of DT was also highlighted. The qualitative analysis also identified various objectives driving DT implementation, from monitoring and control to operations optimization and system behavior prediction. This examination showcased the transformative potential of DTs in the O&G industry, emphasizing the interplay between technology and strategic business objectives.

While our study provides valuable insights, it has limitations, such as the scope of literature reviewed and the rapid pace of technological advancements in DT development. Future work could explore emerging technologies, include more industry case studies, and examine the long-term impacts of DT implementation on operational efficiency, sustainability, and safety. Additionally, as DTs evolve, standardized frameworks and methodologies are needed to guide their development and application across different O&G industry sectors.

The exploration of DTs in the O&G sector represents a frontier of technological innovation poised for significant growth. The ongoing development and refinement of DTs offer a promising avenue for addressing the complex challenges faced by the industry, leading to increased operational efficiency, safety, and sustainability by leveraging real-time data, advanced analytics, and machine learning. This study contributes to the existing knowledge and sets the stage for future research and development efforts in this dynamic and evolving field.

## Figures and Tables

**Table 1:** Criteria for study inclusion and exclusion.

Step	Criteria for Inclusion	Criteria for Exclusion
Title	Studies discussing aspects aligned with digital twins or O&G.	Studies devoid of references to digital twins, O&G, or related terminology.
Abstract	Studies should touch upon themes relevant to our research questions. Relevant secondary studies, such as related SLRs, were also considered.	Studies not strictly fitting the context were shortlisted for subsequent text evaluation. However, those unrelated to our research questions were omitted.
Text	Studies that highlight applications of digital twins in the O&G industry and that directly touch upon our research questions.	Studies lacking demonstrable use cases of digital twins in O&G.

**Table 2:** Main stages and relevant aspects.

Main Stages	Relevant Aspects
Diagnosis and Architecture Design	Specific objectives for the DT must be identified along with functional and non-functional requirements necessary to achieve these objectives. The processes involved in the scope of the problems should be understood and documented, and physical assets such as valves, pipes, and equipment should be digitally represented. Development of DTs involves defining various components and their interactions within the system. This includes components such as contextual data, data processing capabilities, memory capabilities, and simulation capabilities. Architectural design of the digital twin system includes components like the database engine, application engine, visual engine, solution engine, and collaboration engine, which are integrated to create an effective computational system.
Contextual Data Integration and Processing	DTs must encapsulate contextual data to provide a holistic understanding of the operational environment. Data preparation and collection are crucial steps in developing DTs, involving the gathering of operational data, contextual data, and business knowledge. Techniques such as data resampling and balancing are used to handle data imbalances. Machine learning methods, including deep learning, are employed for data-driven solutions, such as anomaly detection and predictive maintenance. These methods involve training virtual models using operational data and deploying them for real-time monitoring and detection.
Modeling and Simulation	Physical models of the system are simulated using software tools to generate numerical experiments. Artificial Neural Networks (ANNs) can be utilized to develop digital twin models, allowing for the representation of complex systems and their relationships between input variables and performance indicators.
Validation and Optimization	Validation of digital twin models involves testing their predictive power and accuracy using real-world data, often through statistical analysis and comparison with existing systems. Optimization techniques, such as multiobjective optimization, are applied to refine digital twin models and improve their performance in achieving specific objectives, such as reducing downtime and increasing efficiency.

**Table 3:** Summary of monitored variables and parameters in different application domains.

Article	Application Domain	Monitored Variables/Parameters
[74]	Asset Monitoring and Maintenance	Equipment health status, KPIs, primary flow rate, inlet temperature, inlet/outlet pressure, inlet conductivity, surge tank level
[71]	Asset Monitoring and Maintenance	LiDAR sensors for pipeline construction attributes
[41]	Asset Monitoring and Maintenance	Load, friction, temperature (sensor-instrumented tribosystems)
[76]	Asset Monitoring and Maintenance	Motion sensors, angle and tension sensors, ADCP for current measurement, fluid monitoring.
[38]	Asset Monitoring and Maintenance	Motion/accelerometers, subaquatic strain gauges, Acoustic Doppler Current Profiler, wave radar, pressure and temperature sensors.
[44]	Asset Monitoring and Maintenance	Pressure and flow line sensors, temperature sensors, scrubber levels, control valve positions, compressor and motor data (vibration, temperature, electrical parameters).
[63]	Asset Monitoring and Maintenance	Pressure and temperature sensors at various process points (bottom hole, wellhead, flowline).
[77]	Asset Monitoring and Maintenance	Pressure, temperature, vibration, flow line sensors, flare line sensors, scrubber levels, valve positions, compressor and engine data
[78]	Asset Monitoring and Maintenance	Sensor data, P&IDs, ERP, depth-based trajectories, and other operational data
[79]	Asset Monitoring and Maintenance	Temperature sensors for real-time data capture to calculate induced thermal stresses.
[37]	Asset Monitoring and Maintenance	Temperature, pressure, flow rates, liquid levels in annulus, leakage rate trends
[47]	Drilling Operations	APWD sensor
[60]	Drilling Operations	Drilling depth, penetration rate, RPM, flow rate, cuttings concentration
[72]	Drilling Operations	High-frequency direction sensors, LWD sensors, vibration sensors
[80]	Drilling Operations	Pressure and temperature data, mud flow data, ROP, well stability, pore pressure data, dynamic torque, and drag simulations
[81]	Drilling Operations	Real-time data focusing on fluid pressure and dynamics (specific sensors not mentioned)
[48]	Drilling Operations	RFID sensors, GIS, Video (parameters not directly mentioned but include drilling parameters, equipment performance, geological analysis)
[82]	Drilling Operations	Torque and drag, hookload, indicators for stuck-pipe events
[73]	Drilling Operations	Triaxial accelerometers, gyroscopes, acoustic modem, and transducer
[83]	Drilling Operations	Well hydraulics, torque and drag, cuttings concentrations and bed formation (not specified sensors)
[43]	Exploration and Reservoir Management	Data from simulator, temperature, and pressure variables
[49]	Exploration and Reservoir Management	IoT-enabled equipment sensors for operational parameters monitoring, including PLCs, SCADA, DCS, ICS.
[46]	Exploration and Reservoir Management	LWD sensors for drilling and exploration management
[61]	Exploration and Reservoir Management	Optical sensors for fluid data in wells
[84]	Exploration and Reservoir Management	Seismic, geological, simulation, and production data integration
[75]	Production Optimization	Digital instruments at well sites and production facilities, Data Historian Server, Real-Time Optimization System (ROS), Specific instrumentation like pressure gauges, multivariable transmitters, Red Eye water cut meters
[65]	Production Optimization	Laser scanning, 360 degrees HD photogrammetry
[85]	Production Optimization	Pressure sensors, SCM data, simulation-based data on fault conditions

**Table 4:** Tools for the digital development of the asset.

Geometric Modeling	Physical Modeling	Rule Modeling
AutoCAD	Hypermesh	ANSYS
UG	Abaqus	Twin Builder
3D Max	ANSYS	3DMax
CATIA	LMS-Samtech	Dymola
SolidWorks	LUSAS	Machining
Maya	ADINA	ADAMS
MeshLab	Nastran	SimuWorks
Twin Builder	AIgor	Recurdyn
FieldTwin	COMSOL Multiphysics	MWorks
InkerCAD	FEPG	Vericut
FreeCAD	Stella	SimulationX
OpenSCAD	MARC	OpenModelica
Inventor Wings 3D	Simulink	Tecnomatix
Onshape	Twin Builder	DELMIA
Meshmixer	AMESim	3DVIA
ProE	ASPEN	Composer
Fusion	HYSYS	
AMESim	SysML	
Autodesk		
LiDAR		

**Table 5:** Metrics and approaches used to evaluate the effectiveness and representational power of digital twins.

Articles	Approaches
[36,87]	Accuracy was mentioned as an evaluation metric.
[51,55,59]	Evaluation was completed involving the concept of KPIs (Key Performance Indicators).
[41,44,77,85,93]	Cross-validation was used to test the accuracy of the trained models.
[94]	Experiments with different datasets were used to show significant improvements in accuracy through evaluation metrics: F1-score and AUC-ROC.
[37,41,61,93,95,97]	Some type of statistical error was employed as a comparative assessment of the model’s performance with the real behavior of the asset, such as RSME (Root Mean Square Error), RESS (Residual Estimated Sum of Squares), and MAE (Mean Absolute Error).
[84]	Direct connection to the database and the “history matching” technique are used to ensure the digital twin is continuously updated with the latest data and that the models are aligned with historical production data.
[70]	Regular optimizations are performed to monitor the gap between potential and actual production.
[64]	Dynamic digital twins feature performance monitoring modules but do not specify which metrics were used.
[37]	A specific module was utilized for validation.
[98]	Validation was mentioned, but it was not specified what DT validation process was used.

**Table 6:** Digital twin solutions.

Digital Twin Software	Company	Application Area	References
Ansys CFX	ANSYS Inc.	Engineering, Manufacturing	[40]
AVEVA	AVEVA Group plc	Industrial	[99,100]
APM; Predix	General Electric	Industrial, Energy	[73]
MindSphere; RTPO	Siemens	Industrial, IoT	[74,101]
3DEXPERIENCE; SIMULIA	Dassault Systèmes	Industrial	[36]
Matlab/Simulink	MathWorks	Engineering, Manufacturing	[43,87,95,102]
FieldTwin	FutureOn	Oil and Gas; Subsea	[89]
Holowells Digital Twin Well	Vertechs	Oil and Gas; Drilling	[45]
INTELIE LIVE	INTELIE	Oil and Gas	[83,100]
iDARE	iDARE	Oil and Gas	[36,69]

## Data Availability

The data are contained within the article.

## Abbreviations

The following abbreviations are used in this manuscript:

DTs	digital twins
O&G	oil and gas
SLR	systematic literature review
IoT	Internet of Things
ANN	Artificial Neural Network
PLS	Partial Least Squares
PCA	Principal Component Analysis
GAN	Generative Adversarial Networks
LSTM	Long Short-Term Memory
CNN	Convolutional Neural Network
ERP	Enterprise Resource Planning
KPI	Key Performance Indicator
RMSE	Root Mean Square Error
MAE	Mean Absolute Error
ROS	Real-Time Optimization System
RFID	Radio Frequency Identification
LWD	Logging While Drilling
RPM	Revolutions Per Minute
ROP	Rate of Penetration
ADCP	Acoustic Doppler Current Profiler
SCADA	Supervisory Control and Data Acquisition
DCS	Distributed Control System
PLC	Programmable Logic Controller
SCM	Supply Chain Management
IIoT	Industrial Internet of Things
MQTT	Message Queuing Telemetry Transport
OPC UA	Open Platform Communications Unified Architecture
REST API	Representational State Transfer Application Programming Interface
LiDAR	Light Detection and Ranging
